# Unlocking Gamete Quality Through Extracellular Vesicles: Emerging Perspectives

**DOI:** 10.3390/biology14020198

**Published:** 2025-02-13

**Authors:** Notsile H. Dlamini, Alessandra Bridi, Juliano Coelho da Silveira, Jean M. Feugang

**Affiliations:** 1 Department of Animal and Dairy Sciences, Mississippi State University, Starkville, MS 39759, USA; nhd30@msstate.edu; 2 University of the West of Santa Catarina, Xanxerê 89820-000, SC, Brazil; alessandra.bridi@unoesc.edu.br; 3 Department of Veterinary Medicine, University of São Paulo, Pirassununga 13635-900, SP, Brazil; julianodasilveira@usp.br

**Keywords:** assisted reproduction, embryo development, fertility, follicular fluid, in vitro culture, nanoparticles, biofluid, seminal plasma, testis

## Abstract

Extracellular vesicles (EVs) are gaining recognition for their essential role in enhancing gamete quality and improving outcomes in assisted reproductive technologies. These nanosized particles, released by cells, carry proteins, lipids, and RNAs, facilitating critical cell communication and offering the potential to enhance gamete maturation and improve fertilization rates. Most research on males has concentrated on seminal plasma, a complex fluid produced by the testes and accessory glands vital in modulating sperm fertility potential. The components of seminal plasma significantly affect sperm functionality, embryo survival, and placental development, making this a prominent area of interest in reproductive biology. The EVs within seminal plasma contribute to maintaining sperm membrane stability, enhancing motility, and promoting capacitation, which may influence the female reproductive tract following mating. In females, EVs have been identified in both the follicular and uterine environments, where effective embryo–maternal communication is crucial. The oviduct epithelium supports gamete transport and early embryonic development, with EVs found in oviductal fluid playing a key role in reproductive processes. These EVs support the embryo’s growth in the nutrient-rich uterine environment. These important studies underscore the significant role of EVs in transporting essential molecular compounds to gametes and embryos, leading to an enhanced understanding and potential manipulation of reproductive processes. This review aims to summarize the current research on the benefits of EVs in gamete manipulation and embryo development, highlighting their promising implications for reproductive health.

## Simple Summary

Research indicates that seminal plasma is essential for creating an optimal environment for spermatozoa, thereby enhancing sperm function and fertility. Effective communication among sperm cells, developing embryos, and maternal tissues is critical for successful pregnancies. Small extracellular vesicles play a significant role in these interactions, contributing to reproductive processes and supporting embryo development. This review underscores the importance of small extracellular vesicles in improving gamete quality in both males and females across various species, highlighting their crucial role in reproductive success.

## 1. Introduction

Gamete quality is a vital component driving the success of the livestock industry, particularly as global demand for animal protein continues to rise. The pig industry, a significant facet of international agriculture, primarily utilizes artificial insemination (AI) as its primary breeding method. By employing fresh, chilled semen, commercial pig centers not only propagate superior genetics but also significantly mitigate the risks of disease transmission [1]. The importance of high-quality semen for effective AI cannot be overstated; selecting boars with outstanding semen quality is crucial for minimizing costs associated with animals not used for semen production [2,3]. Despite notable advancements in precision assessment tools, such as computer-assisted sperm analysis (CASA), the lack of reliable predictive markers for semen quality remains a significant challenge. This gap emphasizes the pressing need for ongoing research and innovation in this field.

In males, most research has focused on seminal plasma (SP), the non-cellular component of semen, employing advanced omics approaches like transcriptomics, metabolomics, and proteomics. However, why concentrate on this fluid that serves to transport sperm? SP is a complex mixture of secretions from the testes, epididymis, and accessory sexual glands. It provides the essential living environment for sperm and is rich in biomolecules, including lipids, proteins, RNA, energy substrates, enzymes, and extracellular vesicles [4]. Importantly, SP is critical in regulating sperm functionality, supporting embryo survival, and facilitating proper placental development, thereby influencing fertility following natural mating and AI [4,5,6]. Notably, SP proteins are essential for maintaining sperm membrane stability, ensuring the sperm’s fertilizing capacity during transport, and modulating both motility and capacitation.

Research on the female reproductive tract has predominantly concentrated on the environments surrounding follicles and the uterus, explicitly examining follicular, oviductal, and uterine fluids. Establishing and maintaining pregnancy in mammals hinges upon effective communication between the embryo and maternal tissues. Oocyte maturation occurs within the preovulatory follicle, followed by oviduct fertilization and early embryonic development during the luteal phase [7]. The oviduct epithelium, comprising ciliated and secretory cells, plays a pivotal role in gamete transport, fertilization [8,9], and embryonic genome activation (EGA) [10].

Extracellular vesicles (EVs) serve as integral mediators of intercellular communication within both male and female reproductive tissues, regulating various physiological processes [8,11]. These vesicles are enriched with bioactive molecules, including microRNAs (miRNAs), messenger RNAs (mRNAs), proteins, and lipids [12,13], which influence several reproductive aspects, such as sperm motility and capacitation, follicular development, oocyte maturation, fertilization, and early embryo development [8,11,14,15,16]. The comprehensive review by De Ávila et al. [17] emphasizes the critical function of EVs as communication entities within follicular [11], oviductal [18], and uterine fluids [19]. Small EVs or exosomes in the oviductal fluid are essential for numerous reproductive processes [20]. In bovine species, the embryo typically reaches the uterus 4 to 5 days post-fertilization [21,22], subsequently developing into a blastocyst [23]. Uterine fluid, abundant in nutrients and EVs, provides vital support for embryonic development and placentation, facilitating crucial intercellular communication for implantation and the establishment of pregnancy [24,25]. These findings illustrate the indispensable role of EVs within the female reproductive tract. Recent research investigates the therapeutic applications of EVs, highlighting their potential roles in drug delivery, diagnostics, and enhancing gamete quality.

A thorough understanding of EVs’ influence on gamete and embryo qualities is crucial for strengthening reproductive health and advancing assisted reproductive technologies (ARTs). Insights into EV-mediated interactions between sperm, egg, and embryo development could lead to the optimization of IVF protocols and the identification of EV-based fertility biomarkers. This knowledge gain offers valuable resources for further genetic selection programs. This review emphasizes the physiological implications of EVs for the functionality of both male and female gametes and embryos.

## 2. Extracellular Vesicle (EV) Characteristics

EVs are membrane-bound substances released by maturing blood reticulocytes into the extracellular space [26]. They are crucial in cell-to-cell communication, facilitating local (autocrine and paracrine) and distant interactions between cells and tissues. These vesicles, encased by a phospholipid bilayer membrane, circulate in body fluids and serve as carriers for proteins, lipids, and RNAs [27].

EVs can originate from the endosomal system or be shed from the plasma membrane of parent cells and tissues [28]. Based on size, they are classified as exosomes (40–120 nm), microvesicles (50–1000 nm), and apoptotic bodies (1–5 μm) [29]. The overlapping size ranges can complicate the differentiation between these subtypes. Exosomes form through the inward budding of endosomal membranes, maturing into multivesicular bodies (MVBs) that release exosomes into the extracellular environment upon fusion with the plasma membrane [27,30]. In contrast, microvesicles are generated by the outward budding of the plasma membrane, while apoptotic bodies emerge from membrane blebbing during programmed cell death [31].

**Figure 1:**
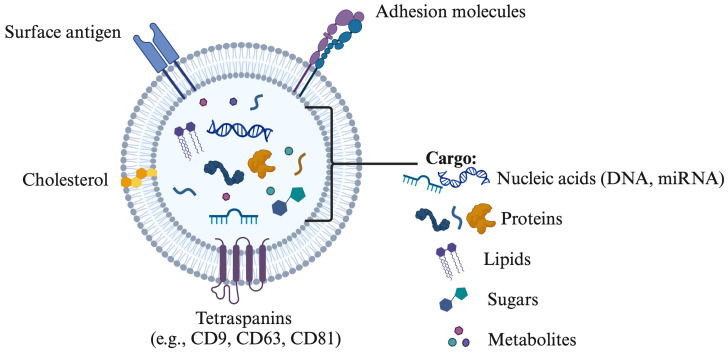
Composition of extracellular vesicles including surface antigen, cholesterol, tetraspanins (CD9, CD63, CD81), nucleic acids (DNA, miRNA), proteins and lipids. The drawing was created using Biorender.com.

The terminology associated with EVs includes terms such as prostasomes, epididymosomes, oviductosomes, and uterosomes, each representing vesicles released by the prostate, epididymis, oviduct, and uterus, respectively [8,32,33,34]. In mammals, EVs play a crucial role in various physiological processes by transporting cell-specific cargo that facilitates communication with target cells while protecting against extracellular degradation [26,35]. The binding of EVs to target cells is influenced by their composition—such as proteins, sphingomyelin, and miRNAs—and the specific receptors on the target cells or tissues (Figure 1). Tetraspanins, including CD9, CD63, CD81, and CD82, are significant membrane proteins abundant in exosomes and serve as key markers for their characterization and isolation [36,37]. Additionally, exosomes contain a range of membrane proteins, signaling molecules, multivesicular body (MVB) proteins, and proteins vital for vesicle trafficking [14].

The investigation of EVs, particularly those from the male and female reproductive tracts, is progressing rapidly due to their potential in therapeutic strategies and biomarker development. This review will examine the roles of EVs derived from seminal plasma and follicular/oviductal fluids, emphasizing their unique populations and functions.

## 3. Extracellular Vesicles (EVs) in the Male Reproductive Tract

### 3.1. Detection

Sperm develop the essential ability to move and fertilize an oocyte during its passage through the epididymis, a process intricately regulated by interactions with seminal fluid during ejaculation [38]. The composition of seminal plasma (SP) is not only complex but also critical for facilitating intense communication through EVs [37]. These EVs are key components of SP, originating from various organs within the male reproductive system, including the testes, epididymis, vas deferens, and accessory glands, such as the prostate and seminal vesicles [6,37]. The existence of EVs in the male reproductive tract was first noted over 50 years ago, with initial observations of vesicle-like structures in rabbit semen [39].

#### 3.1.1. Epididymosomes

In the mid-1980s, Yanagimachi et al. [40] revealed that these EVs, known as epididymosomes, are produced through apocrine secretion, whereby cytoplasmic blebs detach from the epithelial cells lining the epididymis. Epididymosomes constitute a heterogeneous population of vesicles, ranging from 50 to 250 nm, and have been extensively examined across different segments of the epididymis (caput, corpus, and cauda), where they play a vital role in sperm maturation [37].

Epididymosomes play a crucial role in the interaction between sperm and the epididymis by temporarily fusing with sperm to deliver essential proteins such as macrophage migration inhibitory factor (MIF) and P26h/P34H. These proteins are vital for sperm maturation and facilitate binding to the extracellular matrix and the zona pellucida [41,42]. MIF, aldose reductase, and other polyol pathway enzymes significantly enhance sperm motility during epididymal maturation, while type 5 glutathione peroxidase (GPX5) prevents premature acrosome reactions [43,44]. Additionally, the presence of Liprin α3 and the kinase c-Src—proteins associated with the acrosome reaction—within epididymosomes highlights their importance. This suggests that these vesicles are instrumental in the biochemical modifications of ejaculated sperm [45,46], preparing them for successful fertilization.

**Figure 2:**
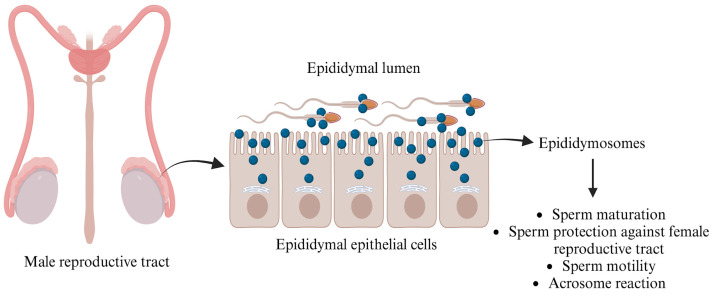
Functions of epididymosomes in the male reproductive tract. The figure is created using BioRender.com and adapted from [53].

Numerous studies have emphasized the essential role that epididymal secreted proteins, particularly those anchored with glycosylphosphatidylinositol (GPI), play in sperm maturation. A notable protein in this context is P26h [47]. Research conducted on hamster spermatozoa indicates that the association of P26h with epididymosomes decreases as sperm travel through the excurrent duct, providing compelling evidence that epididymosomes are critical for acquiring this protein during sperm maturation [48]. In bulls, P25b, the ortholog of P26h, is similarly GPI-anchored to spermatozoa. Investigations have demonstrated that this protein is transferred to sperm collected from the bovine epididymis, enabling these sperm cells to effectively bind to the zona pellucida of oocytes [49]. The importance of epididymosomes goes beyond mere protein transfer; they also transport the transmembrane protein “a disintegrin and metalloproteinase” (ADAM7) to sperm cells (Figure 2), which is vital for successful sperm–oocyte interactions [50]. Moreover, epididymosomes encapsulate small non-coding RNAs (sncRNAs), which are crucial for transferring microRNAs (miRNAs) and transfer RNA-derived RNA fragments (tRFs) to sperm, thus influencing embryo development [51]. After leaving the testes, sperm engage with epididymal EVs, leading to significant changes in miRNA profiles that are important for maturation. For instance, studies reveal that mouse sperm collected from the caput and cauda segments of the epididymis lose 133 miRNAs while gaining 115 miRNAs, respectively [52]. Additionally, sperm incubated with epididymosomes show a significant increase in five specific miRNAs (miR-191, miR-375, miR-467a, miR-467d, and miR-467e), which are present in much higher amounts in epididymosomes compared to sperm that were not exposed to epididymosomes [51]. This evidence underscores the indispensable role of epididymosomes in enhancing sperm functionality and ultimately promoting successful fertilization.

#### 3.1.2. Prostasomes

Prostasomes are prostate-derived vesicles averaging 150 nm in diameter (with a range of 30 to 200 nm) and were first described in human semen in 1978 [54]. They protect spermatozoa from the female immune response within the reproductive tract. They effectively modulate the complement pathway and inhibit the phagocytosis of sperm by monocytes and neutrophils [32]. In addition, prostasomes transport a variety of important proteins [33,55], including signal transduction proteins, guanosine triphosphates, adenosine triphosphates, and prostate-specific markers, such as prostatic acid phosphatase and prostate stem-cell antigen. These proteins serve structural functions and exhibit antimicrobial, antioxidant, and immune-regulatory effects that enhance sperm health [56,57,58]. Furthermore, prostasomes play a significant role in influencing the physiological parameters of ejaculated spermatozoa. Studies have demonstrated their crucial involvement in processes such as capacitation and the acrosome reaction [59], which are vital for successful fertilization. Notably, prostasomes are high in cholesterol and sphingomyelin, which contribute to the fluidity and stability of sperm membranes, particularly in the acidic environment of the vagina [60]. Overall, the diverse functions of prostasomes are designed to ensure that spermatozoa remain in optimal condition and retain their full functional capabilities before encountering the oocyte.

Yet, the precise physiological functions of these vesicles are still under investigation, but numerous studies emphasize their importance in enhancing the recovery of hyperactive motile sperm and improving calcium signaling—both crucial factors in elevating sperm motility [61,62]. Understanding and leveraging these mechanisms could significantly advance our knowledge of male fertility and reproductive health.

### 3.2. Role of Seminal Plasma-EVs (SP-EVs) in Modulating Gamete Function

Most SP-EVs’ effects are reported in spermatozoa, known to be transcriptionally and translationally silent following spermatogenesis, underscoring EVs’ crucial role in the male reproductive tract concerning post-testicular sperm maturation and fertilizing capacity. SP-EVs significantly influence various sperm functions through their binding and fusion with ejaculated sperm. The epididymis facilitates sperm maturation via sequential interactions with intraluminal fluids that change sperm morphology [38]. These interactions prompt essential structural and compositional changes, including adjustments in phospholipid composition, cholesterol/phospholipid ratio modifications, increased total negative charges, and alterations to surface proteins. As a result, spermatozoa acquire critical functions, including progressive motility, zona pellucida binding, and fusion with the oocyte’s plasma membrane [37,38]. Seminal plasma contains the highest concentration of EVs among bodily fluids, with approximately ~10^12^ particles per ejaculate. SP-EVs interact with spermatozoa to support sperm development [63]. The specific binding sites of SP-EVs correlate directly with their functions: EVs engaging with the sperm head enhance capacitation, activate the acrosomal reaction, and improve oocyte binding capacity; conversely, those bound to the mid-piece and central piece of the tail regulate mitochondrial activity, energy metabolism, and overall motility [5].

A substantial body of research underscores the pivotal role of SP-EVs in promoting sperm functionality and enhancing reproductive success. For instance, studies conducted by Arienti et al. [64] have indicated that EVs derived from human seminal plasma markedly influence sperm function, with EVs from normozoospermic men significantly enhancing sperm motility and facilitating capacitation when co-cultured with ejaculated sperm [65]. In porcine models, Du et al. [14] noted that SP-EVs considerably improved sperm motility during the incubation of extended sperm. Additionally, boar sperm subjected to higher concentrations of exosomes demonstrated increased motility, prolonged survival, enhanced membrane integrity, and elevated total antioxidant capacity following thawing while simultaneously preventing premature capacitation [14]. In other species, such as sheep, spermatozoa in seminal plasma EVs are associated with proteins involved in vesicle biogenesis, metabolism, and membrane adhesion and remodeling functions [66]. Furthermore, analyses of boar SP-EVs have revealed that the predominant proteins comprise structural proteins (notably actin), enzymes, intracellular ion channels, and sperm adhesins [4], thereby emphasizing the multifaceted contributions of SP-EVs in optimizing sperm functionality and promoting reproductive success.

Extracellular vesicles are integral to transferring small non-coding RNAs, which carry various biological functions depending on their source [67]. This remarkable ability enables EVs to deliver specific microRNAs (miRNAs) to spermatozoa, playing a significant role in post-testicular sperm function. Notable miRNAs found in SP-EVs, such as ssc-miR-148a, ssc-miR-10a-5p, and ssc-miR-10b, are essential for spermatogenesis and fertility [68,69]. Interestingly, most small RNAs identified in SP-EVs from Yorkshire boars are miRNAs [70]. Analysis of expression levels shows that 83.71% of these miRNAs are at low levels, while 16 are highly expressed in SP-EVs [71], suggesting a selective mechanism for sorting into exosomes [72]. Additionally, studies indicate that boar SP-EVs contain differentially expressed miRNAs associated with low sperm cryotolerance [73] and divergent motility [74]. This suggests the potential of these miRNAs as biomarkers to aid in predicting sperm viability and quality during cryopreservation and AI dose preparation, respectively.

In mammals, miRNAs found in SP-EVs are essential for assessing semen quality and fertility [75,76]. A notable study by Barcelo et al. (2018) demonstrated that miR-31-5p is a highly sensitive and specific biomarker for azoospermia based on its expression in human seminal plasma exosomes derived from azoospermic individuals [77]. The study also identified miR-210-3p as highly expressed in the seminal exosomes of patients with varicocele, further emphasizing the diagnostic potential of these miRNAs.

In porcine studies, researchers [74] conducted a comparative analysis of SP-EVs from Duroc semen of varying quality, identifying key miRNAs such as ssc-miR-205, ssc-miR-493-5p, and ssc-miR-378b-3p, which are directly linked to boar sperm quality (good vs. poor). Furthermore, high-throughput sequencing was employed to examine the miRNA profiles of boars, revealing critical differences in low- and high-motility sperm across Landrace and Yorkshire breeds [78]. Six miRNAs—ssc-miR-122-5p, ssc-miR-486, ssc-miR-451, ssc-miR-345-3p, ssc-miR-362, and ssc-miR-500-5p—were identified as being differentially expressed in both breeds. These miRNAs play significant roles in essential biological processes, including gene expression regulation and critical signaling pathways such as PI3K-Akt, MAPK, and Ras, highlighting their potential as non-invasive markers for predicting boar semen motility. These signaling pathways regulate sperm motility by inhibiting PI3K expression in semen [78]. Specifically, ssc-miR-362, ssc-miR-486, and ssc-miR-122-5p enhance sperm motility by activating vital genes associated with the PI3K-Akt signaling pathway, including PIK3R1, PRKCA, PIK3CB, IGF1, LAMC1, and FOXO3. Additionally, ssc-miR-362 and ssc-miR-486 may influence the MAPK pathway through interactions with PRKCA, IGF1, and STK4 [78,79], underscoring their potential significance as biomarkers in reproductive science and as non-invasive biomarkers for predicting sperm motility in boars.

In avians, SP-EVs are found to be significantly more abundant in the semen of fertile roosters compared to their sub-fertile counterparts. This distinction is critical, as studies suggest that transferring SP-EVs from fertile roosters to sub-fertile ones can effectively enhance sperm motility [80]. Additionally, the differentially expressed miRNAs present in SP-EVs derived from duck semen are enriched in essential pathways related to sperm functions, such as the ErbB signaling pathway, glycometabolism, and ECM-receptor interactions, all of which are vital for regulating sperm motility [81]. Beyond their miRNA content, SP-EVs can synthesize ATP through glycolysis, further promoting mitochondrial metabolism and motility in sperm [82]. It is noteworthy that the interactions between sperm and EVs are pH-dependent [5]: The alkaline pH of prostatic secretions inhibits early EV/sperm fusion, while the acidic environment of the vagina facilitates it. These studies provide compelling evidence for SP-EVs’ role in enhancing sperm function; however, further research is needed to fully understand the specific contributions of their various components.

## 4. Extracellular Vesicles (EVs) in the Female Reproductive Tract

EVs play a crucial role in regulating female reproductive biology, being released from various regions within the female reproductive tract, including follicles, the oviductal and uterine epithelium, as well as the embryo/conceptus [8,15,83].

### 4.1. EVs in Follicular Fluid

More than 30 studies have explored these EVs’ biological functions and molecular cargo, which encompass both exosomes and microvesicles found within the follicular fluid (FF). The process of folliculogenesis, which involves the growth and maturation of ovarian follicles, relies significantly on effective cell-to-cell communication among theca, granulosa, and cumulus cells. This communication is facilitated by constituents in FF, including hormones, proteins, and anti-apoptotic factors. Notably, the cargo profile of EVs undergoes substantial changes throughout the stages of follicular development, as FF-EVs transport essential RNAs, miRNAs, and proteins that influence the transcriptional activity of recipient cells [11,83].

Research spanning equine, bovine, and human subjects has confirmed the presence and importance of EVs in FF. For example, studies in horses have identified specific miRNAs within exosomes and microvesicles in FF that regulate key pathways, including transforming growth factor beta (TGF-β), wingless (WNT), and mitogen-activated protein kinase (MAPK). These pathways are essential to folliculogenesis, encompassing processes such as follicular development, granulosa cell proliferation, cumulus expansion, steroidogenesis, and meiosis, as well as mammalian embryo mitosis [11,83]. In bovine research, a significant correlation was observed between elevated expression of EV-miRNAs around immature oocytes and increased transcriptional activity during oocyte growth [83]. This suggests that the uptake of exosomes by follicular cells is linked to higher levels of miRNAs. Additionally, EV-miRNAs found in small follicles, indicative of early antral follicle growth, are associated with genes involved in cell proliferation pathways. In contrast, those in larger follicles, representing later stages of growth, are connected to genes related to inflammatory response pathways [84]. In humans, the miRNAs present in FF-EVs primarily participate in pathways such as WNT, epidermal growth factor receptor (ErbB), MAPK, and TGF-β, which regulate crucial processes like follicular development, meiotic resumption, and ovulation [85].

Studies in bovines revealed that the stage of the estrous cycle markedly influences the composition of miRNA and proteins within FF-EVs, primarily attributed to fluctuations in progesterone (P4) levels in the FF, resulting from the corpus luteum. Interestingly, FF-EVs with low P4 concentrations display a unique miRNA profile that regulates essential biological pathways, including MAPK, RNA transport, Hippo signaling, cell cycle processes, FoxO signaling, oocyte meiosis, and TGF-beta signaling [17]. Additionally, a thorough study using an in vivo model demonstrated that the protein content of FF-EVs varies depending on the follicle’s proximity to the corpus luteum, specifically whether it is in the ovary ipsilateral or contralateral to the CL [86]. Notably, FF-EVs from the ovary ipsilateral to the CL, characterized by higher intrafollicular P4 levels, exhibit an increased abundance of proteins linked to the retraction of actin filament-based membrane projections, such as transzonal projections (TZPs). In contrast, FF-EVs from the contralateral ovary, showing lower P4 concentrations, have reduced protein abundance [86]. These findings highlight the crucial role of FF-EVs in regulating key molecular pathways essential for folliculogenesis, oocyte development, and cumulus cell function.

### 4.2. Extracellular Vesicles (EVs) in Embryo–Maternal Interactions

The endometrial epithelium significantly contributes to communication with the embryo during implantation by releasing EVs, responsible for transferring vital signaling miRNAs and adhesion molecules to the blastocyst [59]. Exosomal markers CD9 and CD63 located on the apical surfaces of human endometrial epithelial cells suggest that the endometrial epithelium is a source of EVs in the uterine cavity generated via the apocrine pathway. These EVs carry proteins essential for cell migration, adhesion, angiogenesis, and focal adhesion kinase (FAK) signaling, fostering a supportive embryo implantation microenvironment [87].

#### 4.2.1. Evidence from the Maternal Side

Oviductal EVs: Protein analyses of bovine oviduct epithelial cells (BOECs) indicate that oviductal EVs contain proteins such as oviductal glycoprotein (OVGP), heat shock protein A8 (HSPA8), and myosin 9 (MYH9), which play essential roles in fertilization, early pregnancy development, and zona pellucida maturation [18,88]. However, the BOECs used in the in vitro system did not present OVGP [18], suggesting that the in vitro environment can alter the composition of oviductal EVs. An in vivo model has shown that EVs derived from oviductal fluid in pregnant cows 120 h after ovulation induction display a unique miRNA profile compared to those from non-pregnant cows. Specifically, eight miRNAs (bta-miR-126-5p, bta-miR-129, bta-miR-140, bta-miR-188, bta-miR-219, bta-miR-345-3p, bta-miR-4523, and bta-miR-760-3p) were found to be upregulated in the pregnant group and are predicted to influence crucial signaling pathways, such as PI3K-Akt, MAPK, and mTOR [89]. Furthermore, EVs derived from oviductal fluid in the isthmus are associated with higher embryo survival rates following vitrification in cattle, with increased expression of Aquaporin 3 (AQP3) contributing to enhanced membrane fluidity [90].

Uterine EVs: They are detected in the uterine lumen of various species, originating from both the luminal and glandular epithelium of the uterus [91]. EVs derived from uterine lumen fluid play a significant role in conceptus elongation [19] and communication between the embryo and the maternal environment, influencing various biological processes, such as the differentiation of trophoblast binucleate cells, the induction of apoptosis in immune cells, and the promotion of cellular proliferation, which are essential elements for establishing uterine receptivity to conceptus implantation [92]. These beneficial effects are attributed to the EVs content in interferon tau (IFNT) and prostaglandin contents [93], while a study demonstrated the participation of uterine EVs of pregnant sheep in RNA transfer, further emphasizing the uterine EVs role in embryo–endometrium interactions [94].

#### 4.2.2. Evidence from the Embryo Side

A recent study utilizing an ex vivo model with endometrial and luteal explants has revealed that EVs secreted by embryos and endometrial cells exhibit varying miRNA content based on the origin of the embryo (in vivo vs. in vitro). These EVs play a role in modulating mRNA and miRNA profiles in luteal tissues, indicating their involvement in embryo–maternal interactions during the establishment of pregnancy [95]. Additionally, both day-7 to day-16 in vivo- and in vitro-derived embryos release EVs containing distinct miRNA that are predicted to influence pathways in the endometrium and corpus luteum (CL) associated with the maternal recognition of pregnancy [96]. When the bovine endometrium is exposed to these EVs, it undergoes global transcriptomic changes, underscoring the endometrium’s capability as a biosensor in cattle, which is partly mediated by its response to the unique miRNA cargo from conceptus-derived EVs during the establishment of pregnancy.

**Figure 3:**
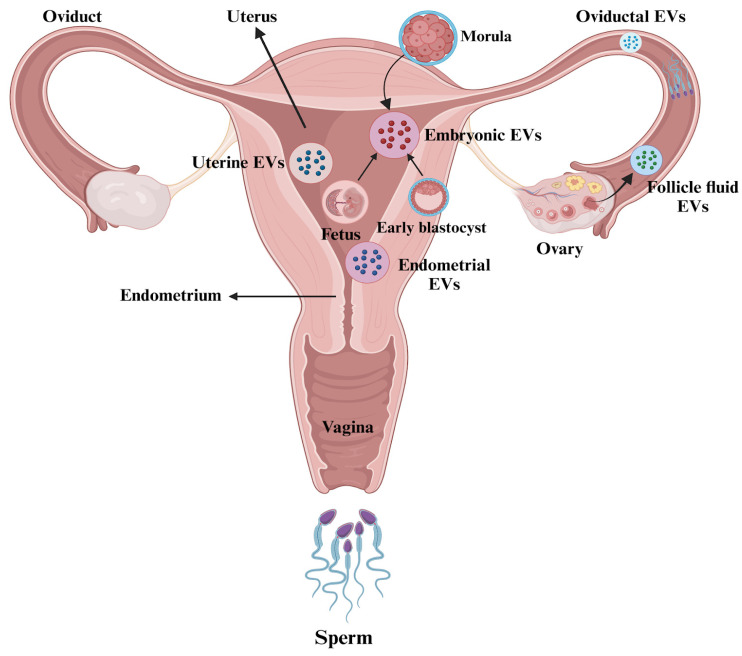
Extracellular vesicles (EVs) in the female reproductive tract. The figure is created using BioRender.com and adapted from [97].

Overall, these findings highlight the significant functional roles of EVs in follicular growth, the modulation of embryo development, and the interactions between the embryo and the oviduct, all of which can enhance fertilization and embryo quality. It is critical to consider ways to improve the application of EVs in ART. Figure 3 illustrates the presence of EVs in the female reproductive tract.

### 4.3. Impacts of EVs on Sperm Function

EVs in the female reproductive tract support sperm function, which is essential for various biological processes in female reproduction. Effective cell-to-cell communication within this microenvironment is crucial for developing oocytes and follicles, potentially facilitated by the secretion and uptake of EVs [83]. EVs in follicular fluid facilitate the transfer of miRNAs and proteins between cells. Sohel et al. [83] reported an upregulation of numerous miRNAs in the exosome fraction of follicular fluid, suggesting increased transcriptional activity during oocyte growth. These exosomes regulate several signaling pathways, including ubiquitin-mediated, neurotrophin, MAPK, and insulin signaling, important for ovarian follicular development and other developmental processes. Moreover, exosomes derived from follicular fluid influence the expression of ACVR1 and ID2 in cultured granulosa cells by transferring mRNAs, proteins, and miRNAs, thereby affecting follicular development [98].

In oviductal fluid across various species, EVs contain proteins and RNAs (mRNA, miRNA, rRNA, tRNA) critical for fertilization processes [34]. Research has shown that EVs from porcine oviductal fluid enhance the efficiency of in vitro fertilization by improving parameters such as sperm viability, motility, capacitation, and sperm binding while reducing the likelihood of polyspermy. These beneficial effects are attributed to proteins such as OVGP1, MYH9, ANXA1, 4, and 5 [99,100]. Additionally, oviductal EVs have been found to improve sperm viability when combined with frozen semen, with the degree of effectiveness varying according to the stage of the estrous cycle [100].

In domestic cats, it was found that oviductal EVs enhance sperm motility and acrosomal integrity when pre-incubated with fresh epididymal spermatozoa. This effect is mediated by proteins such as OVGP1, HSP70, HSP90, CD9, CCT8, and CCT7 [101]. Additionally, research demonstrated that adding oviductal EVs improves in vitro embryo development in pigs and influences the embryonic transcriptome, thereby highlighting the maternal impact at the molecular level [102]. Furthermore, EVs found in uterine fluid play crucial roles in somatic cell nuclear transfer (SCNT) embryo development during the early luteal phase [103]. Recent studies have also isolated EVs from human uterine fluid, showing their rapid interaction with sperm, which promotes capacitation, enhances motility, and induces the acrosome reaction [104].

During the early stages of pregnancy in pigs, EVs derived from uterine luminal fluid (ULF) have been shown to contain miRNAs that regulate EV transportation and conceptus development [105]. These ULF EVs regulate the proliferation and migration of embryonic trophoblast cells, which are essential for successful implantation. Overall, these studies underscore the functional roles of EVs in the female reproductive system and elucidate their significance in reproductive processes such as sperm function, gamete maturation, fertilization, implantation, and embryo development.

### 4.4. Impacts of EVs on Embryo–Oviduct–Endometrium Interactions

There is a growing interest in the role of EVs in gamete and embryo–maternal communication, particularly concerning their implications in embryo–oviduct interactions. The oviduct facilitates gamete maturation, sperm transport, capacitation, fertilization, and early embryo development. Oviductal EVs (oEVs) and embryo-secreted EVs (eEVs) have emerged as key participants in the bidirectional communication between the oviduct and the embryo [106]. Numerous studies have highlighted this interaction, demonstrating that oEVs are internalized by embryos at the blastocyst stage in vitro [18], as well as by oviductal epithelial cells [107], enhancing embryo implantation, development, and overall quality [18,20,108]. Analyses of these vesicles in mice have identified specific miRNAs such as miR-143-3p, miR-22-3p, and miR-34c-5p, which are crucial for the first cleavage and implantation of mouse embryos [109]. In bovine models, EVs are enriched with mRNAs that include genes for histone methyltransferases, histone demethylases, and the DNA methyltransferase gene (DNMT1), all of which are associated with embryo development, cell proliferation, and epigenetic regulation [34]. Furthermore, miR-449a, which is upregulated in oEVs during the post-ovulatory period, has been linked to various factors contributing to infertility in both men and women. Target analysis of miR-449a revealed a network of genes involved in embryo development, angiogenesis, and responses to oxidative stress, suggesting its significance in achieving a successful pregnancy [34].

#### 4.4.1. Embryo-Secreted EVs

Exosomes derived from the ovine conceptus trophectoderm at 15 and 17 days of gestation contain several crucial proteins, including interferon tau (IFNT), macrophage-capping protein (CAPG), and aldo-keto reductase family 1, member B1 (AKR1B1) [110]. Treatment of endometrial epithelial cells with these exosomes during the pre-implantation period (P17 UFs) resulted in an upregulation of apoptosis-related genes, such as BCL2-associated X, apoptosis regulator (BAX), caspase 3 (CASP3), tumor necrosis factor (TNFA), and tumor protein P53 (TP53) transcripts [111]. Furthermore, exosomes from the post-implantation phase (P20 and P22 UFs) stimulated the expression of adhesion molecules, notably vascular cell adhesion molecule 1 (VCAM1) mRNA [112]. These findings highlight the critical role of exosome-mediated changes in the uterine environment for the attachment and development of the conceptus. Conversely, exosomes from porcine trophectoderm and aortic endothelial cells contain miRNAs that are predicted to influence angiogenesis and placental development pathways, indicating their significant role in communication between the conceptus and the maternal endometrium, ultimately impacting the establishment of pregnancy [113].

#### 4.4.2. Embryo-Secreted EVs Influenced by Culture Conditions

The secretion of embryo-secreted extracellular vesicles (eEVs) varies according to the biotechnology utilized for embryo production. A study by [111] observed differences in the size and concentration of eEVs between parthenogenetic (PA) and in vitro fertilization (IVF) embryos. Recent research also suggests that the concentration of eEVs is influenced by the embryo’s developmental stage, sex, and the in vitro culture (IVC) conditions. Moreover, in vitro-produced bovine embryos were found to secrete more eEVs than their in vivo counterparts from day 7 to day 9 of development. However, the size of the vesicles remained comparable between the two groups [96]. Notably, fourteen miRNAs were upregulated, while two were downregulated in EVs from in vivo embryos compared to those from in vitro embryos. These miRNAs are predicted to regulate key pathways involved in pregnancy establishment in cattle, including oxytocin signaling, MAPK, Ras, glycerophospholipid metabolism, lysine degradation, HIF-1, and the WNT pathway [96]. Additionally, a higher concentration of eEVs was observed in day 7 bovine embryos compared to day 3 under 20% oxygen tension, indicating a positive correlation with successful pregnancy outcomes [114]. Conversely, a lower concentration of eEVs in culture media was associated with the failure to achieve pregnancies, in contrast to embryos with higher eEV concentrations [115].

Furthermore, a study utilizing a co-culture system to examine paracrine communication between porcine embryos—specifically parthenogenetic (PA) embryos and cloned embryos produced through somatic cell nuclear transfer (SCNT)—demonstrated that co-culturing PA embryos significantly enhanced the in vitro development of cloned embryos. This enhancement was linked to the upregulation of mRNA expression for key pluripotency markers, such as OCT4, KLF4, and NANOG, which are contained within EVs [116]. EVs derived from oviductal and uterine fluids closely mimic the physiological conditions present during the in vitro culture (IVC) of bovine embryos. A recent study found that incorporating these EVs into IVC systems did not significantly alter embryo development rates; however, it markedly improved blastocyst quality. Notable enhancements included higher survival rates following vitrification and warming, increased total cell numbers, and improved lipid metabolism [117]. These findings highlight the crucial role of EVs from embryos of different origins—oviductal fluid (OF) and uterine fluid (UF)—as key modulators of embryo quality in both swine and bovine IVC systems. These observations underscore the crucial roles of embryonic EVs in embryo development and inter-embryo communication. Table 1 illustrates the multifunctional roles of male and female EVs in mammalian female reproduction.

## 5. Possible Therapeutic Applications of Extracellular Vesicles in Treating Infertility

Infertility is a widespread reproductive condition that increasingly affects both human health and the livestock industry on a global scale [136]. EVs have emerged as significant mediators of intercellular communication, playing essential roles in various physiological processes related to mammalian reproduction [137,138]. Numerous studies highlight the potential of EVs as therapeutic agents and diagnostic tools in ARTs, which could revolutionize reproductive health [139]. Recent investigations in mares have identified multiple miRNAs derived from FF-EVs associated with distinct stages of follicle development and variations between breeding and non-breeding seasons [140,141]. These specific miRNA profiles may be potential biomarkers to enhance fertility outcomes through improved ART practices. Research has also focused on the use of EVs as biomarkers for various fertility disorders, including polycystic ovary syndrome (PCOS), endometriosis, Asherman’s syndrome, and preeclampsia [142,143,144]. For example, EVs derived from human follicular fluid contain small RNAs that may play a role in the pathogenesis of PCOS, thus serving as molecular biomarkers for its diagnosis [145]. Furthermore, one study found that platelet-derived EVs were elevated in the plasma of women with PCOS, correlating with increased serum testosterone levels [146].

EVs have shown considerable potential in addressing primary ovarian insufficiency (POI). Research indicates that EV-derived miRNAs are associated with the progression and treatment of POI. For instance, a study highlighted that EVs from human adipose mesenchymal stem cells (hAMSCs) can mitigate ovarian function damage through the SMAD signaling pathway in mouse models of POI [147]. Similarly, EVs from human umbilical cord mesenchymal stem cells (hUMSCs), which encapsulate miR-17-5p, have demonstrated therapeutic potential by suppressing PARP1, γH2AX, and XRCC6 via SIRT7 inhibition [148]. Additionally, research has shown that endometriosis stromal cells can enhance angiogenesis in vitro through the EVs they secrete, further influencing angiogenic processes by regulating endothelial and stromal cells [149,150].

Regarding male fertility, numerous studies have identified exosome-related proteins, particularly exosomal annexin II, as potential biomarkers for male reproductive disorders [151,152]. Moreover, the prostaglandin D2 synthase (PTGDS) gene, which codes for an enzyme, was found to be significantly lower in patients with obstructive azoospermia compared to those with non-obstructive azoospermia (NOA), underlining its potential as a diagnostic biomarker for obstructive azoospermia [153]. Beyond the male reproductive tract, EVs derived from normozoospermic men, including those post-vasectomy, have been shown to significantly enhance sperm motility. In contrast, EVs from asthenozoospermic men appear to impair motility, highlighting their potential implications for male fertility and therapeutic applications [154].

Small EVs or exosomes have gained significant attention as therapeutic drug delivery systems due to their stability, low immunogenicity, and potential for bioengineering [155]. Recent advancements in nanotechnology have facilitated the encapsulation of therapeutic agents, such as miRNAs and small molecules, within EVs and their modification with various ligands for targeted delivery [155,156]. While research into the role of EVs in reproductive disorders is progressing rapidly, further investigation is essential, and these findings open up promising opportunities for clinical applications.

## 6. Conclusions

This review underscores the crucial role of extracellular vesicles (EVs) as emerging tools for identifying specific molecular markers linked to mammalian reproduction. EVs transport diverse biomolecules essential for cellular communication and gamete function. They are instrumental in various reproductive processes, including sperm motility, maturation, fertilization, embryo development, and implantation. The impact of EVs derived from in vivo, in vitro, or cloned embryos highlights their potential as biomarkers for evaluating embryo quality and pregnancy potential. However, despite their significance, further mechanistic studies are necessary to gain a comprehensive understanding of the physiological roles of EVs in both male and female reproductive systems, their origins, and the specific alterations in their cargo within target tissues. These insights could elucidate the molecular mechanisms governing EVs and pave the way for innovative therapeutic strategies to improve assisted reproductive techniques.

## Figures and Tables

**Table 1:** Examples of multifunctional roles of male and female extracellular vesicles in mammalian female reproduction.

Source of Extracellular Vesicles (EVs)	Species	Functional Role in the Female Reproduction
Seminal plasma	Human	Low fertilization rates were associated with reduced expression of galectin-3 in seminal EVs [118]. Proteins from EVs expressed in women with recurrent pregnancy loss (RPL) led to defects in paternal gene expression and embryo development [119].
	Mice	Sperm supplemented with seminal extracellular vesicles (EVs) lacking Arrestin Domain Containing 4 (Arrdc4-/-) were unable to fertilize oocytes [120]. Age-related changes in the content of seminal extracellular vesicles (EVs) from young males increased the embryo implantation rate [121].
	Porcine	Sperm incubated with seminal plasma-derived extracellular vesicles (EVs) showed an increased acrosome reaction compared to sperm in the control group [122]. Co-culture porcine embryos significantly enhanced the in vitro development of cloned embryos [116]. Seminal fluid extracellular vesicles (EVs) act as modulators of vacuolar-type H+-ATPase, contributing to immune activation at conception [123].
Oviductal fluid	Mice	Adding oviductal EVs derived from donor oviductal fluid to the culture media improved embryo quality [108]. Oviductal EVs reduced reactive oxygen species in rabbit embryos, thereby providing protection against molecular damage [124].
	Porcine	Incubating porcine oocytes with oviductal fluid EVs improved resistance to proteolytic degradation and decreased zona pellucida binding, thereby reducing polyspermy [125].
	Bovine	Oviductal fluid-derived EVs from the isthmus resulted in the greatest bovine embryo survival rate after vitrification, compared to EVs from the ampulla or the absence of EVs [90]. EVs secreted by bovine oviduct epithelial cells (BOECs) in vivo expressed proteins such as oviductal glycoprotein (OVGP), heat shock protein A8 (HSPA8), and myosin 9 (MYH9), associated with fertilization, early pregnancy development, and zona pellucida maturation [18]. Incubation with frozen oviductal EVs increased the expression of genes such as Y box-binding protein 1 (YBX1) in bovine embryos, involved in the embryo developmental process [126].
Follicular fluid	Porcine	Transcriptome analysis of porcine follicular fluid extracellular vesicles (FF-EVs) revealed that mRNAs isolated from FF-EVs are highly enriched in genes associated with PI3K-AKT and MAPK pathways, as well as metabolic processes such as lipid metabolism, glycolysis, and cholesterol biosynthesis involved in follicular development and oocyte competence [127].
	Bovine	EVs from small follicles added during in vitro maturation and in vitro culture improved blastocyst rates as well as modulated epigenetic marks and gene expression [128]. EVs released from FF from small follicles (3–5 mm in diameter) and large follicles (>9 mm in diameter) promote cumulus expansion during in vitro maturation [16]. EVs from small and large follicles increased granulosa cell proliferation in a stage-specific manner through Src, PI3K/Akt, and MAPK [129]. FF-EVs directly target and modulate pathways involved in follicular growth and oocyte maturation, including the Wnt signaling pathway, transforming growth factor beta (TGF-β), mitogen-activated protein kinase (MAPK), and epidermal growth factor receptor (ErbB) [83]. EVs derived from FF with low P4 concentrations exhibited a distinct miRNA profile compared to those from FF with higher P4 levels [17,130]. These miRNAs are predicted to regulate pathways such as MAPK, RNA transport, Hippo, cell cycle, FoxO, oocyte meiosis, and TGF-beta signaling [17]. Moreover, cumulus cells supplemented with EVs from low P4 FF showed upregulated genes associated with oocyte development, immune responses, and the Notch signaling pathway [17].
Placental fluid	Human	In vitro, human placental cytotrophoblast cells responded to hypoxia by altering the bioactivity of EVs, thereby promoting the migration of extravillous trophoblasts into the decidua and myometrium, and establishing placental perfusion [112].
Endometrium EVs	Mice	EVs collected from the endometrium during the pre-receptive phase, receptive phase, and the time of implantation exhibited differential expression of miRNAs, including miR-34c-5p, which regulates growth arrest-specific gene 1 (GAS1), a gene associated with embryo implantation [131].
Embryonic EVs	Mice	Embryos co-cultured with EVs demonstrated a higher blastocyst formation rate and a reduced apoptotic rate in pregnant mice compared to embryos cultured without EVs [132].
	Porcine	Supplementation with exosomes secreted by SCNT embryos in the culture medium increased the blastocyst rate, total cell numbers, ICM/TE ratio, and transcript levels of OCT-4 compared to SCNT embryos without supplementation [133].
	Bovine	Individual embryos cultivated in bovine serum albumin medium supplemented with EVs derived from conditioned embryo culture medium showed significantly lower apoptosis and increased blastocyst rates [107]. Embryos produced in vivo or in vitro from day 7 to day 9 can secrete extracellular vesicles containing different miRNA predicted to modulate endometrium and CL pathways related to maternal recognition of pregnancy [96].
Uterine luminal fluid	Ovine	Labeled EVs isolated from the uterine fluid, transferred to ovine trophectodermal cells in vitro within 2 h of co-incubation, resulted in both increased proliferation and release of interferon τ (the ovine pregnancy recognition signal) in a dose-dependent manner [15].
	Bovine	Addition of EVs collected from uterine flushing around the time of implantation led to the downregulation of transcripts associated with the immune system in endometrial epithelial cells [134]. Bovine uterus-derived exosomes used during in vitro SCNT embryo culture improved blastocyst formation, increased total cell number, elevated interferon tau levels, and reduced expression levels of BAX and HSP70 [135]. An ex vivo model based on endometrial and luteal explants demonstrated that EVs secreted by a single day-7 embryo and endometrial cells have different miRNA content depending on embryo origin (in vivo or in vitro) and that these EVs alter the mRNAs and miRNAs profile in luteal tissue [95].

## Data Availability

There are no generated data.
